# ﻿Subjective assessment of working conditions on watercrafts

**DOI:** 10.13075/ijomeh.1896.02685

**Published:** 2026

**Authors:** Dariusz Pleban, Łukasz Kapica, Julia Idczak, Magdalena Warszewska-Makuch, Witold Sygocki

**Affiliations:** 1 Central Institute for Labour Protection – National Research Institute, Department of Physical Hazards, Warsaw, Poland; 2 Central Institute for Labour Protection – National Research Institute, Department of Ergonomics, Warsaw, Poland; 3 Central Institute for Labour Protection – National Research Institute, Scientific Information, Warsaw, Poland

**Keywords:** job satisfaction, risk factors, surveys and questionnaires, workplace, occupational health, working conditions

## Abstract

**Objectives:**

Vessels constitute a unique type of workplace, primarily due to their operation in non-standard external environments and the spatial constraints inherent to their design. As a result, onboard working conditions play a critical role in ensuring the health and safety of crew members. This study aimed to evaluate the subjective assessment of working conditions among seafarers and to analyze the relationship between perceived environmental burdens and job satisfaction.

**Material and Methods:**

The study was conducted on a group of 300 employees working on inland, port, coastal, and Baltic Sea vessels. A questionnaire survey was used to collect data on the subjective evaluation of working conditions and the perceived intensity of environmental factors such as noise, vibrations, and microclimate. Correlations between these factors and job satisfaction were analyzed.

**Results:**

The assessment revealed that 84% of surveyed seafarers rated their overall working conditions positively. However, environmental burdens were prevalent: noise (82%), vibrations (71%), and microclimate (65%) were identified as the most common nuisances. A negative correlation was observed between the perception of environmental burdens and job satisfaction.

**Conclusions:**

Although the general assessment of working conditions on vessels was positive, noise, vibrations, and microclimate remain significant issues affecting the well-being and job satisfaction of seafarers. Preventive measures aimed at mitigating these burdens may contribute to improving occupational health and safety onboard vessels.

## Highlights

- The majority of seafarers rate their working conditions positively.
- Stronger environmental burden perception is linked to lower job satisfaction.
- No significant differences found between navigation types in burden perception.
- Noise and vibrations are the most burdensome environmental factors for seafarers.

## INTRODUCTION

The global economy is significantly influenced by maritime and inland waterway transport. Water transport is considered the most cost-effective and environmentally friendly mode of transportation. Its structure and operational mechanisms offer several advantages, including low transportation costs and minimal energy consumption [1]. However, it also presents notable disadvantages, such as slow transit speed and high dependency on meteorological conditions. Another critical issue is the high incidence of vessel-related accidents, particularly given that human error significantly contributes to maritime safety risks, with nearly 70% of incidents attributed to such mistakes. These errors frequently result from cognitive, perceptual, and psycho-behavioral factors [2,3], often linked to poor working conditions and occupational stress. Additionally, sleep disturbances caused by rotational shifts and noise on vessels represent 1 of the primary stressors among seafarers leading to increased rates of injuries and operational failures due to lapses in attention or negligence during work hours [3,4]. Moreover, it should be emphasized that shift work involves night-time duties performed under conditions of limited visibility, which can contribute to a high accident rate and an increased risk of health problems [5]. Furthermore, this work-related fatigue contributes to miscommunication among crew members, which is also commonly recognized as a high-risk factor, along with failures in bridge resource management (BRM). The latter are often associated with insufficient BRM training, which remains inadequate and requires substantial improvement and expansion [6–8].

As previously outlined vessels, including inland, port, coastal, and Baltic types, represent unique workplaces. They operate in an unique environment and are characterized by spatial limitations, which are often a source of job dissatisfaction [9]. As noted by Marczak [10], the functioning of ship crew memberst – including both work and rest – is influenced by several factors, such as working conditions, vessel movement (rocking), changing weather, and the organization of tasks performed at various times of the day. Work on vessels is typically arranged in rotational shifts, such as 4 h on/8 h off or 6 h on/6 h off work/rest schedules, and duties may vary depending on the time of day, with night shifts involving additional challenges such as previously discussed limited visibility, increased risk of sleep disturbances, and restricted access to medical assistance in the event of an accident. Additionally, as mentioned in Febriyanto et al. [11] noise pollution plays a significant role in deteriorating the working conditions of watercraft personnel, since, according to the definition of the Statistics Poland, working conditions are understood as a set of factors occurring in the work environment, resulting from the work process and factors related to the performance of work [12]. This definition broadly outlines the overall conditions that must be ensured for workers. Moreover, Polish occupational safety standards are aligned with international norms, including the International Labour Organization's (ILO) Occupational Safety and Health Framework. Even though working conditions are only defined by Statistics Poland, the ILO defines decent work as the aspirations of people in their working lives. It involves opportunities for work that is productive and delivers a fair income, security in the workplace and social protection for all, better prospects for personal development and social integration, freedom for people to express their concerns, organize and participate in the decisions that affect their lives and equality of opportunity and treatment for all women and men [13]. Moreover, International Maritime Organization Standards of Training, Certification and Watchkeeping for Seafarers (IMO STCW) Code A-VIII/1 set minimum rest time requirements to ensure that seafarers maintain maximum focus during working hours. The IMO STCW Code A/VIII/1 apply primarily to Baltic navigation as well as port and coastal navigation, and are not legally required in the case of inland navigation.

On the other hand, it has to be emphasized that the working environment comprises physical (e.g., lighting, noise, microclimate), chemical (e.g., toxic substances), and biological (e.g., bacteria) factors present both in the immediate workplace (e.g., factory hall, workstations) and in the surrounding area. Carter [14] provides a detailed description of the most important and hazardous factors present on ships. A major challenge in limiting these undesirable factors arises particularly when considering seafarers that are required to live and work on board vessels for extended periods throughout the year. This unique situation makes the working environment simultaneously a living environment, which is one of the primary sources of job dissatisfaction [9,15,16]. Furthermore, long working hours and prolonged separation from family constitute additional stressors [17]. Additionally, according to literature data, noise and vibrations as well as temperature and humidity play a crucial role among the negative factors of the working environment on vessels [16,18–21]. Existing literature [22–24] highlights the considerable impact of occupational stress among seafarers, as well as the elevated risk of depression resulting from traumatic incidents and their consequences.

To provide a thorough analysis encompassing key parameters related to the subjective evaluation of working conditions, a survey was developed. Surveys are a widely used and effective method for gaining insight into human behavior and perceptions. Numerous studies worldwide have employed surveys to emphasize the significance of working conditions in ensuring a healthy and safe work environment.

The aim of this article is to evaluate the subjective assessment of working conditions among seafarers and to analyze how perceived environmental burdens relate to their overall well-being. In this context, overall well-being is understood as a multidimensional concept that encompasses job satisfaction, psychological well-being, and self-rated general health. Since these factors are closely interrelated, understanding how seafarers perceive the burden of their working environment provides valuable insight into the determinants of their occupational well-being and the potential risks associated with prolonged exposure to unfavorable working conditions. The sample consisted of 100 workers from each type of navigation (inland, port and coastal, and Baltic) to ensure a comprehensive comparison across different maritime sectors. The respondents were selected to reflect a diverse group and serve as a representative sample of the broader seafaring population.

This article is divided into sections. First, it provides a detailed description of the methodology used in the survey, with an emphasis on practical considerations and tailored solutions appropriate to the nature of the study and the respondents. The initial sections also outline the characteristics of the participants, including gender distribution, age groups, occupations, job tenure, and working hours. Following this introductory and methodological overview, the results are presented, and the article concludes with a discussion and final conclusions.

## MATERIAL AND METHODS

### Survey design

Regular surveys by the European Foundation for the Improvement of Living and Working Conditions (Eurofound), Dublin, highlight the importance of assessing occupational risks. These assessments should include both objective and subjective methods. Subjective risk assessments are influenced by individual employee characteristics, psychological aspects of the work environment, and the perceived level of occupational risk. Although indirect, these assessments provide valuable insights into workers' exposure to occupational hazards and their potential impacts on health and well-being. The relevance of subjective evaluations is closely aligned with the World Health Organization's definition of health: “Health is not merely the absence of disease or infirmity, but a state of complete physical, mental and social well-being” [25].

In view of the above, a comprehensive research methodology was adopted to assess working conditions on vessels. In addition to environmental analyses, the methodology included a survey focused on the individual perception of working conditions on board. Conducting the survey required the development of a dedicated questionnaire, which is described in detail in Pleban et al. [26]. The form comprised 110 questions, including 10 socio-demographic and employment-related items, and 49 questions concerning the characteristics of working conditions, particularly environmental risks and nuisances. It also included questions about respondents' general well-being and health status, as well as their perceptions of noise, vibrations, electric lighting, type of workspace, and microclimate. Additional items addressed the technical equipment of the vessels. The questionnaire included both ordinal-scale (categorical) and open-ended questions. Furthermore, 2 standardized psychological instruments were incorporated, including the *Copenhagen Psychosocial Questionnaire* (COPSOQ III) [27], and the *General Health Questionnaire* (GHQ-28) [28]:

– COPSOQ III was used to assess selected aspects of psychosocial working conditions. Specifically, the job satisfaction and self-rated health subscales were applied. Self-rated health status was measured with a single item, as this is a 1-item measure, internal consistency could not be assessed. In the present study, McDonald's ω coefficient were acceptable for job satisfaction subscale: 0.870
– GHQ-28 was used to assess the mental health status of adults experiencing short- or long-term psychological distress, which may result from life challenges or deteriorating mental health. The total score reflects the respondent's overall psychological well-being. In the present study, the total GHQ-28 score was used as an indicator of psychological difficulties. McDonald's ω coefficient for the scale was 0.916, indicating high internal consistency.

The study also included items from an original questionnaire developed by the authors. This part of the survey focused on participants' general perceptions of working conditions on watercraft, including physical aspects. The first question was: “How do you assess your working conditions?” Respondents chose 1 of the following 5 options, which were ranked 1–5 for the purpose of numerical analysis: “very good,” “good,” “average,” “bad,” “very bad.”

The second question referred to specific environmental factors and was phrased as follows: “Considering the past 12 months during which you worked on a watercraft, please indicate whether each of the following working environment factors was burdensome: noise, vibrations, electric lighting, optical radiation (ultraviolet radiation – UVR, infrared radiation – IR), microclimate, mechanical factors causing injury (e.g., moving parts of equipment, slippery or uneven surfaces), dust and chemicals, odor.” For each factor, respondents could choose from the following response options: “not burdensome,” “burdensome,” “not applicable.”

Additionally, for respondents who identified a given factor as burdensome, the level of burden for each working environment factor was assessed using a line scale ranging from 0 (“not burdensome”) to 10 (“extremely burdensome”). The scale included the following verbal descriptors: 0–1 – “not burdensome,” 2–4 – “slightly burdensome,” 5–6 – “moderately burdensome,” 7–8 – “very burdensome,” and 9–10 – “extremely burdensome.”

The study was conducted using the diagnostic survey method, specifically employing the paper and pencil interview (PAPI) technique, i.e., a face-to-face interview based on a paper questionnaire. Prior to participation, respondents were informed about the objectives of the study and were required to give their consent. The interviewer then read the questions aloud and recorded the responses. Respondents retained the right to withdraw from the study at any time or to skip any questions they chose not to answer. Due to the specific nature of the PAPI method, responses were entered into the database both during and after the data collection phase. The survey was conducted in accordance with the standards and values promoted by the Interviewer Work Quality Control Program [29]. This Polish quality assurance framework, developed by the Polish Association of Public Opinion and Market Research Firms (Organizacja Firm Badania Opinii i Rynku – OFBOR), is one of the most important national initiatives in the field. It ensures that survey implementation aligns with international standards, including those set by European Society for Opinion and Marketing Research (ESOMAR).

The methodology for assessing working conditions on vessels (which included both the survey and environmental studies), as well as the developed questionnaire, were positively evaluated by the Bioethics Committee of the Witold Chodzko Institute of Rural Medicine in Lublin, Poland (Resolution No. 20/2023, dated September 29, 2023).

### Data analysis

As part of the process, a comprehensive field verification was carried out, covering 100% of the collected material. The following issues were subject to quality control:

– the collected data were reviewed for the percentage of questions in which respondents either refused to answer or indicated uncertainty (e.g., “I do not know”/“it is difficult to say”). The most significant gaps were found in questions that did not provide the option to refuse or select “I do not know”/“it is difficult to say” which resulted in incomplete data. To address this, missing values were supplemented with the median of all other responses to the same question. This approach allowed the inclusion of these observations in further analyses;
– logical coherence check that involved verifying the consistency of respondents' answers. The relationship between responses to different questions was analyzed to identify any unreliable data.

For responses to the survey questions, percentage data were presented, indicating the proportion of answers for each point on the scale relative to the total number of responses. To compare the distribution of nominal variables between groups, the χ^2^ test was used. For comparisons involving ordinal variables, the Kruskal-Wallis test was applied. Associations between ordinal variables were analyzed using Kendall's τ_b_ correlation coefficient.

To compare quantitative variables between navigation types, a 1-way ANOVA was performed. When statistically significant effects were observed, Tukey's *post hoc* tests were conducted.

The significance level was set at α = 0.05, and all statistical tests were 2-tailed. Group difference tests were supplemented with effect size measures [30,31]: Cramér's V for the χ^2^ test, ε^2^ for the Kruskal-Wallis test, partial η^2^ for ANOVA, and Cohen's d for *post hoc* pairwise comparisons.

All statistical analyses were conducted using Jamovi (2.3.28) based on R language.

### Participants

At the outset of the study, it was assumed that the vessels on which the respondents were employed met specific criteria: they had to be equipped with an engine, enclosed space, and electric lighting. Moreover, the work performed by the participants had to take place while the vessel was in motion.

The survey was conducted among individuals working on vessels. Based on their self-reported area of employment, participants were classified into 3 groups of navigations: inland, port and coastal, Baltic Sea.

The total number of participants was 300, including 67 women (22%) and 233 men (78%), which were significantly more (χ^2^(5) = 216.44, p < 0.001). The sampling procedure was purposive: to enable meaningful comparisons between navigation types, approximately equal group sizes were intentionally established for each category.

The sample size was determined *a priori* to ensure adequate statistical power for between-group comparisons. According to power analyses performed in G*Power 3.1.9.7 (α = 0.05, k = 3 groups), a total sample of 246 for the 1-way ANOVA and 146 for the χ^2^ tests was required to detect effects of medium size (f = 0.20, V = 0.20) with 80% power, confirming that the final sample of 300 participants provided sufficient power for all main analyses. The gender distribution was approximately consistent across all 3 groups of seafarers (Table 1).

**Table 1.** Gender, age, job position, and length of service of employees working on inland, port, coastal, and Baltic Sea vessels, Poland, September–October 2023

Variable	Participants (N = 300)			
	total	inland navigation (N = 100)	port and coastal navigation (N = 100)	Baltic navigation (N = 100)
Gender [%]				
male	78	80	82	71
female	22	20	18	29
Age [%]				
<25 years	2	3	3	1
25–35 years	24	20	23	28
36–45 years	35	42	29	34
46–55 years	32	26	39	32
56–65 years	6	7	5	5
>65 years	1	2	1	0
Job position [%]				
bridge crew	37	41	49	21
repair and maintenance services	19	16	13	27
cook/kitchen assistant	15	25	9	10
passenger service	11	9	6	19
onboard food service	9	8	11	9
fisherman	3	0	8	2
cleaner	2	0	3	4
warehouse keeper	1	0	0	3
other	3	1	1	5
Service time [years]				
M±SD	9.99±7.60	9.44±7.75	10.6±8.33	9.97±6.69
Me	8	8	8	10
min.–max	<1–40	1–37	<1–40	1–34

No significant difference in gender distribution was observed between the groups of seafarers (χ^2^(5) = 3.96, p = 0.138, V = 0.12). The survey also considered the age profiles of the participants. Table 1 presents the age distribution of the respondents. The 2 largest age groups in the research sample were respondents aged 36–45 years (35%) and 46–55 years (32%). The least represented groups were participants <25 years (2%) of age and those >65 years (only 1%). Similarly, there was no significant difference in age between the groups (χ^2^(2) = 0.82, p = 0.663, ε^2^ = 0.003).

The survey included individuals employed in various positions on board vessels. Among all respondents, the largest group consisted of bridge crew members (37%), followed by those working in repair and maintenance services (19%). This distribution is consistent across all subgroups of seafarers – namely, inland, port and coastal, and Baltic Sea navigation. Other notable occupational groups include kitchen staff in inland navigation (25%), passenger service in Baltic navigation (19%) and onboard food service personnel in port and coastal navigation (11%). Detailed information on the respondents' workplace positions is presented in Table 1.

There were no significant differences in the length of service among the different types of navigation, as summarized in Table 1 (χ^2^(2) = 1.49, p = 0.474, ε^2^ = 0.005). On average, respondents had worked on vessels for 10 years.

## RESULTS

The analysis of responses to the question “How do you assess your working conditions?,” which addressed the overall evaluation of working conditions (“very good,” “good,” “average,” “bad,” “very bad”), indicates that the vast majority of surveyed seafarers (84%) rated their working conditions as either “very good” (33%) or “good” (51%). In contrast, 15% of respondents assessed their working conditions as “average,” while only 1% rated them as “bad” (Table 2).

**Table 2.** Outcome of the overall assessment of working conditions on watercraft with Kruskal-Wallis between groups comparison test, Poland, September–October 2023

Variable	Participants (N = 300)												χ^2^(2)	p	ε^2^
	total			inland navigation (N = 100)			port and coastal navigation (N = 100)			Baltic navigation (N = 100)					
	%	M	Me	%	M	Me	%	M	Me	%	M	Me			
Rating category		1.84	2.00		1.90	2.00		1.83	2.00		1.80	2.00	1.19	0.552	0.004
very good (1)	32.7			28.0			36.0			34.0					
good (2)	51.3			55.0			46.0			53.0					
averaged (3)	15.0			16.0			17.0			12.0					
bad (4)	1.0			1.0			1.0			1.0					
very bad (5)	0			0			0			0					

The Kruskal-Wallis test results indicated no statistically significant differences among the 3 types of watercraft concerning responses on working conditions.

When responding to the second question, “Considering the past 12 months during which you worked on a watercraft, please indicate whether each of the following working environment factors was burdensome: noise, vibrations, electric lighting, optical radiation (UVR, IR), microclimate, mechanical factors causing injury (e.g., moving parts of equipment, slippery or uneven surfaces), dust and chemicals, odor,” the surveyed seafarers assessed their working conditions in relation to specific environmental factors (Table 3). The analysis of the responses indicates that noise is the most commonly reported nuisance factor on watercraft – 82% of respondents declared having experienced its burdensome effects over the past 12 months. The majority of them rated noise as moderately burdensome (37%) or slightly burdensome (35%). Vibration is the second most commonly reported nuisance factor on watercraft. In total, 71% of respondents declared experiencing its burdensome effects. Among this group, the majority rated vibrations as “slightly burdensome” (35%) or “moderately burdensome” (27%), while 9% indicated that vibrations were “very burdensome” or “extremely burdensome.”

**Table 3.** Subjective assessment of environmental working conditions with results of χ^2^ test among employees working on inland, port, coastal, and Baltic Sea vessels, Poland, September–October 2023

Factor	Participants (N = 300) [%]												χ^2^(2)	p	V
	total			inland navigation (N = 100)			port and coastal navigation (N = 100)			Baltic navigation (N = 100)					
	n.b.	b.	n.a.	n.b.	b.	n.a.	n.b.	b.	n.a.	n.b.	b.	n.a.			
Noise	18	82	0	21	79	0	7	92	1	25	75	0	12.1	0.002	0.201
Vibrations	27	71	2	27	71	2	22	75	3	32	68	0	2.15	0.342	0.085
Electric lightning	42	58	0	35	65	0	48	51	1	43	57	0	3.76	0.153	0.112
Microclimate	33	65	2	44	54	2	27	72	1	29	70	1	8.18	0.017	0.166
Optical radiation (ultraviolet and infrareded radiation)	52	40	8	49	50	1	52	39	9	55	32	13	3.58	0.167	0.114
Mechanical factors that cause injuries	44	50	6	38	54	8	41	56	3	52	41	7	4.86	0.088	0.132
Dusts and chemicals	56	32	12	53	34	13	54	32	14	62	30	8	0.864	0.649	0.057
Odor	52	39	9	45	42	13	48	44	8	64	30	6	6.57	0.038	0.155
Different	73	5	22	72	9	19	76	5	19	71	2	27	4.32	0.115	0.136

χ^2^ – statistic of the χ^2^ test; V – Cramer's V (effect size for χ^2^ test).

n.b. – not burdensome; b. – burdensome; n.a. – not applicable.

Other commonly reported nuisance factors include:

– microclimate – reported as burdensome by 65% of respondents, with more than half of them (36%) describing it as “slightly burdensome”;
– electric lighting – identified as a nuisance by 58% of respondents, of whom the majority (34%) also rated it as “slightly burdensome”;
– mechanical factors causing injuries (e.g., moving parts, uneven or slippery surfaces) – experienced as burdensome by 50% of respondents. In this group, more than half (31%) rated the nuisance as “slight,” while 44% of all respondents reported no burden at all related to this factor.

The list of the most common nuisance factors on vessels is completed by 3 additional elements: optical radiation (UVR, IR), odor, and dust and chemical substances. Nuisance caused by each of these factors was reported by 40%, 39%, and 32% of respondents, respectively. At the same time, 29% of employees experiencing UVR and IR exposure, 25% of those affected by odor, and 21% exposed to dust and chemical substances rated it as “slightly burdensome.”

A χ^2^ test revealed statistically significant differences in the distribution of responses regarding the perception of noise as a nuisance across navigation types (p < 0.01). Notably, the highest proportion of respondents reporting noise nuisance was found among those employed in port and coastal shipping – as many as 92% of respondents. In comparison, 79% of inland waterway workers and 75% of Baltic seafarers identified noise as a nuisance factor. A similar trend was observed in the assessment of vibrations. Again, the largest group experiencing this nuisance was port and coastal workers (75%), followed by inland waterway workers (71%) and Baltic seafarers (68%). In this case, however, the comparison result was not statistically significant. Statistical analysis also revealed significant differences (p < 0.05) in the distribution of responses concerning the burdensomeness of microclimate and odor. Microclimate was most frequently reported as burdensome by seafarers engaged in port and coastal navigation (72%), followed by those working in Baltic waters (70%) and inland navigation (54%). In the case of odor, the highest proportion of complaints was noted among port and coastal workers (44%), compared to 42% among inland waterway employees and 30% among Baltic seafarers.

The next step of the study aimed to determine the impact of the most burdensome environmental factors on working conditions and overall well-being. This assessment was conducted in 2 ways. First, participants were asked directly whether specific environmental factors (i.e., noise, vibrations, lighting, and microclimate) affect their health and well-being — the results of this subjective evaluation are presented in Table 4. Second, correlations were calculated between the perceived burdensomeness of the factors (as reported in Table 3) and 3 indicators: psychological well-being (measured with the GHQ-28), general health, and job satisfaction (both assessed with the COPSOQ III). The majority of surveyed employees indicated that the previously mentioned environmental factors (i.e., noise, vibrations, lighting, and microclimate) do not significantly affect their health and well-being, as presented in Table 4. In the case of noise, 36% of respondents perceived such an impact, while for vibrations and electric lighting, only 17% and 13%, respectively, reported such effects. Meanwhile, 1 in 4 respondents (25%) recognized the impact of microclimate on their health and well-being. It is worth noting that perceptions of noise as a factor affecting health and well-being vary between navigation types. Half of the port and coastal workers declared that noise affects their lives, compared to <1 in 4 (23%) of inland navigation workers and almost 1 in 3 (35%) of Baltic navigation workers.

**Table 4.** Subjective assessment of the impact of work environment factors on health and well-being among employees working on inland, port, coastal, and Baltic Sea vessels, Poland, September–October 2023

Factor	Participants (N = 300) [%]				χ^2^(4)	p	V
	total	inland navigation (N = 100)	port and coastal navigation (N = 100)	Baltic navigation (N = 100)			
Noise	36	23	51	35	18.81	<0.001	0.177
Microclimate	25	11	39	24	21.30	<0.001	0.189
Vibrations	17	16	19	15	1.54	0.819	0.051
Electric lightning	13	8	13	19	5.54	0.236	0.096

χ^2^ – statistic of the χ^2^ test; V – Cramer's V (effect size for χ^2^ test).

The type of navigation also differentiates perceptions of the microclimate's impact on well-being, but the correlation is low (Cramér's V = 0.189). This effect was more frequently reported by employees in port and coastal navigation (39%) and Baltic navigation (24%).

Overall, the analysis suggests that port and coastal navigation workers are the most affected by environmental factors.

Table 5 presents a comparison of seafarer groups (by type of navigation) in terms of psychological well-being (GHQ-28), self-rated general health (COPSOQ III), and job satisfaction (COPSOQ III).

**Table 5.** Differences in psychological well-being (*General Health Questionnaire –* GHQ-28), self-rated general health and job satisfaction (*Copenhagen Psychosocial Questionnaire* – COPSOQ III) among employees working on inland, port, coastal, and Baltic Sea vessels, between types of navigation, Poland, September–October 2023

Tool	Score (M±SD)			F(2, 297)	p	η^2^_p_
	inland navigation	port and coastal navigation	Baltic navigation			
GHQ-28^a^	42.4±7.82	43.1±9.08	42.6±6.96	0.175	0.840	0.001
Self-rated health (COPSOQ III)	67.3±17.7	75.0±19.1	73.3±17.9	4.97	0.008	0.032
Job satisfaction (COPSOQ III)	73.9±16.2	72.9±16.9	72.9±16.9	0.119	0.888	0.001

a Higher GHQ-28 scores indicate more severe psychological distress or poorer mental health status.

No statistically significant differences were found for psychological well-being or job satisfaction. However, a significant difference emerged for self-rated general health. *Post hoc* analyses revealed that inland navigation crews reported significantly lower levels of general health compared to those working in port and coastal navigation (p_Tukey_ = 0.008, d = 0.425). All other pairwise differences were not statistically significant (p > 0.05).

The results, presented in Table 6, show that greater perceived burdensomeness of noise, microclimate, and vibrations is significantly associated with lower job satisfaction. The strongest negative correlation was found between noise and job satisfaction, followed by microclimate and vibrations. Additionally, electric lighting also showed a weaker but statistically significant negative correlation with job satisfaction.

**Table 6.** Correlations (Kendall's τ_b_) between the perceived burdensomeness of environmental factors and psychological well-being (*General Health Questionnaire* – GHQ-28), self-rated general health and job satisfaction (*Copenhagen Psychosocial Questionnaire* – COPSOQ III) among employees (N = 300) working on inland, port, coastal, and Baltic Sea vessels, Poland, September–October 2023

Factor	GHQ-28^a^		Self-rated health (COPSOQ III)		Job satisfaction (COPSOQ III)	
	τ_b_	p	τ_b_	p	τ_b_	p
Noise	0.081	0.070	–0.224	<0.001	–0.356	<0.001
Microclimate	0.060	0.186	–0.119	0.020	–0.238	<0.001
Vibrations	0.058	0.193	–0.236	<0.001	–0.222	<0.001
Electric lightening	0.019	0.675	–0.069	0.180	–0.148	0.006

a Higher GHQ-28 scores indicate more severe psychological distress or poorer mental health status. Positive correlations thus suggest that greater perceived burdensomeness of a factor is associated with worse psychological well-being.

In terms of general health, negative correlations were found between the burdensomeness of noise, microclimate, and vibrations, indicating that individuals who rated these factors as more burdensome also reported poorer general health. The association between electric lighting and general health was weaker and not statistically significant.

When it comes to psychological well-being, the correlations with GHQ-28 scores were weaker and not statistically significant. Only the association between noise and psychological distress approached significance.

These findings suggest that the subjective perception of certain environmental factors as burdensome – particularly noise and microclimate – may have a greater impact on employees' job satisfaction than on their general or mental health status.

## DISCUSSION

The survey method for assessing subjective evaluations of working conditions is a commonly applied approach in scientific studies. It enables the analysis of individuals' subjective perception of physical factors and their impact on human life. Such research has been conducted among seafarers in various European countries, including Croatia [16], Germany [32], and Sweden [33], as well as in Asian countries like South Korea [34], or in multinational contexts as presented in Akamangwa [35]. To complement the general data collected in other countries, this article presents data on the subjective assessment of working conditions among Polish seafarers employed on inland, port and coastal, as well as Baltic vessels. The data are based on a survey conducted among 300 employees (100 from each navigation type), including 67 women and 233 men. The findings show that the majority of surveyed seafarers (84%) rated their working conditions as “good” or “very good” (51% and 33%, respectively). In contrast, 15% assessed their conditions as “average,” and only 1% described them as “bad.” The highest percentage of positive ratings was recorded among employees in Baltic navigation, where 87% of respondents assessed their working conditions as “good” (53%) or “very good” (34%).

The article also examines the perceived burden of environmental factors present in the workplace. The most frequently reported burdensome environmental factors on vessels were noise (82%) and vibrations (71%), which is consistent with the findings of Forsell et al. [33] where noise was identified as the most commonly reported work-related problem. In shows that only 58% of respondents considered noise pollution to be a significant environmental issue, whereas Jo et al. [34] reports that almost 55% of respondents regarded noise as a hazardous element of the work environment. Moreover, the data presented in Forsell et al. [33] indicate that, on average, 70% of respondents identified noise as a work-related problem, while approx. 50% considered vibrations to be problematic.

Other frequently indicated nuisance factors included microclimate (65%), electric lighting (58%), and mechanical factors causing injuries (50%). In contrast, a majority of employees reported no significant nuisance from dust and chemical substances (68%) or odor (61%) during the performance of their duties.

Most of the differences between inland, port and coastal, and Baltic navigation were not statistically significant. This suggests that, regardless of the navigation type, seafarers perceive their working conditions in a relatively similar way.

Moreover, most respondents indicated that the key physical factors in the work environment – noise, vibrations, electric lighting, and microclimate – although considered burdensome, were perceived by the majority as having no significant effect on their health or well-being. This may reflect individual differences in coping strategies, acceptance of challenging working conditions as normal in the maritime sector, or limited self-awareness regarding these issues. Nevertheless, correlational analysis indicated that a stronger perception of environmental burdensomeness was linked to lower levels of job satisfaction.

This study is not without limitations. First, the reliance on self-report questionnaires may have introduced biases related to subjective perceptions or socially desirable responding – especially considering that part of the data was collected during face-to-face interviews, which might have influenced the openness of some participants. Second, the cross-sectional design limits the ability to draw causal inferences between the analyzed variables. Since no statistically significant relationships were found between perceived environmental burdens and job satisfaction, no conclusions can be drawn regarding the direction or nature of potential associations between these variables. Moreover, this design does not allow for the exploration of temporal dynamics in psychosocial factors, well-being, or work-related experiences – for instance, across different voyage stages, rotations, or shifts.

Third, although the sample was deliberately balanced across 3 groups of seafarers, it was selected through a purposive sampling strategy, which may limit the generalizability of the findings to the broader maritime workforce.

Finally, while the survey comprehensively assessed seafarers' perceptions of environmental working conditions, it did not cover certain key aspects such as work intensity, duration, mode of work, and rest conditions. These factors are known to have a significant impact on both physical and mental health, as well as overall job satisfaction. The exclusion of these variables represents an important limitation; however, they are planned to be examined in the follow-up phase of this research, which will build upon the findings presented here and provide a more universal understanding of the determinants of seafarers' occupational well-being.

Future studies should therefore adopt longitudinal and diary study designs to capture short-term dynamics and longer-term changes in employees' well-being and work experiences. The inclusion of objective indicators – such as physiological measures, work schedules, or company records – along with a more diverse and representative sampling strategy, would strengthen the robustness, validity, and practical applicability of future results.

## CONCLUSIONS

The aim of this article was to evaluate the subjective assessment of working conditions among seafarers and to analyze how perceived environmental burdens relate to their overall well-being. The findings indicate that the majority of Polish seafarers across all navigation sectors perceive their working conditions positively, with 84% rating them as good or very good. Notably, the highest satisfaction was reported by employees in Baltic navigation.

The study also identified noise and vibrations as the most commonly perceived burdensome environmental factors, consistent across navigation types. However, despite recognizing these factors as nuisances, most respondents did not report significant negative impacts on their health or well-being, which may suggest adaptation or limited awareness of potential risks. Importantly, the data showed that stronger perceptions of environmental burdensomeness were associated with lower job satisfaction.

Overall, the study successfully fulfilled its aim by providing a detailed and comparative insight into the subjective working conditions of Polish seafarers across different maritime sectors. These results contribute valuable knowledge to the understanding of occupational environments in the maritime industry and highlight areas for further investigation, especially concerning the health implications of environmental stressors. Future research should build on these findings by incorporating longitudinal designs and objective health measures to deepen understanding of how working conditions affect seafarers' well-being over time.

## References

[1] Maternová A, Materna M, Dávid A. Revealing causal factors influencing sustainable and safe navigation in Central Europe. Sustainability. 2022;14(4):2231. 10.3390/su14042231.

[2] Maternová A, Materna M, Dávid A, Török A, Švábová L. Human error analysis and fatality prediction in maritime accidents. J Mar Sci Eng. 2023;11(12):2287. 10.3390/jmse11122287.

[3] Kim SJ, Jeon TY, Lee YC. Impact of ship noise on seafarers' sleep disturbances and daily activities: An analysis of fatigue increase and maritime accident risk through a survey. Appl Sci (Basel). 2024;14(9):3757. 10.3390/app14093757.

[4] Tait JL, Chambers TP, Tait RS, Main LC. Impact of shift work on sleep and fatigue in maritime pilots. Ergonomics [Internet]. 2021;64(7):856–68. 10.1080/00140139.2021.1882705. 33523762

[5] Arendt J, Middleton B, Williams P, Francis G, Luke C. Sleep and circadian phase in a ship's crew. J Biol Rhythms. 2006;21(3):214–21. 10.1177/0748730405285278. 16731661

[6] Campaniço Cavaleiro S, Gomes C, Lopes MP. Bridge resource management: Training for the minimisation of human error in the military naval context. J Navig. 2020;73(5):1146–58. 10.1017/S0373463320000235.

[7] Shi K, Fan S, Weng J, Yang Z. Seafarer competency analysis: Data-driven model in restricted waters using Bayesian networks. Ocean Eng [Internet]. 2024;311(Pt 2):119001. 10.1016/j.oceaneng.2024.119001.

[8] Ikram AD, Hasugian S, Soeharto S. Analyze the implementation of bridge resource management to avoid dangerous situations on vessel. Dinamika Bahari. 2023;4(2):32–43.

[9] Slišković A, Penezić Z. Descriptive study of job satisfaction and job dissatisfaction in a sample of Croatian seafarers. Int Marit Health. 2015;66(2):97–105. 10.5603/IMH.2015.0023. 26119680

[10] Marczak E. [Human safety on vessels in accordance with actual regulations]. Zesz Nauk. 2009;18(1890):97–104. Polish.

[11] Febriyanto K, Guedes JCC, Das Neves Correia Mourão LJR, Amalia N, Prasetyo BPA, Rahman FF. A critical assessment of occupational noise exposure and relevant variables for hearing disturbances: A study among watercraft personnel. Univ J Public Health. 2024;12(5):811–9. 10.13189/ujph.2024.120503.

[12] [Working conditions in 2023]. Warsaw: Statistics Poland; 2024. Polish.

[13] International Labour Organization [Internet]. Geneva: ILO; 2022 [cited 2025 December 19]. Decent work in nature-based solutions 2022. Available from: https://www.ilo.org/sites/default/files/2024-04/Decent%20Work%20in%20Nature-based%20Solutions%202022.pdf.

[14] Carter T, Jepsen JR. Exposures and health effects at sea: report on the NIVA course: Maritime occupational medicine, exposures and health effects at sea. Int Marit Health. 2014;65(3):114–21. 10.5603/IMH.2014.0024. 25471159

[15] Pleban D, Kowalski P, Zając J. Assessment of noise and vibration annoyance and other physical factors of working conditions on vessels by means surveys – a research method. In: Proceedings of the 52nd International Congress and Exposition on Noise Control Engineering; 2023 Aug 20–23; Chiba, Japan. Institute of Noise Control Engineering – USA (INCE-USA); 2023. p. 853–7.

[16] Vukić L, Mihanović V, Fredianelli L, Plazibat V. Seafarers' perception and attitudes towards noise emission on board ships. Int J Environ Res Public Health. 2021;18(12):6671. 10.3390/ijerph18126671. 34205743 PMC8296330

[17] Jensen HJ, Oldenburg M. Objective and subjective measures to assess stress among seafarers. Int Marit Health. 2021;72(1):49–54. 10.5603/IMH.2021.0007. 33829473

[18] Krystosik-Gromadzińska A. [Occupational safety during inland shipping factual and legal state and *de lege ferenda* remarks]. Bezpieczeństwo Pr Nauk Prakt. 2019;574(7):8–11. Polish. 10.5604/01.3001.0013.2785.

[19] Picu L, Picu M, Rusu E. An investigation into the health risks associated with the noise and vibrations on board of a boat: A case study on the Danube River. J Mar Sci Eng. 2019;7(8):247. 10.3390/jmse7080258.

[20] Turan O, Helvacioglu I, Insel M, Khalid H, Kurt R. Crew noise exposure on board ships and comparative study of applicable standards. Ships Offshore Struct. 2011;6(4):323–38. 10.1080/17445302.2010.514716.

[21] Orosa JA, Oliveira AC. Assessment of work-related risk criteria onboard a ship as an aid to designing its onboard environment. J Mar Sci Technol. 2010;15(1):16–22. 10.1007/s00773-009-0067-0.

[22] Jensen HJ, Oldenburg M. Objective and subjective measures to assess stress among seafarers. Int Marit Health. 2021;72(1):49–54. 10.5603/IMH.2021.0007. 33829473

[23] Pia JVP, Galam R, Bartuseviciene I. Regulating seafarers' welfare: An examination of the protection of Filipino seafarers' well-being through a legal analysis of the POEA-standard employment contract. Int Marit Health. 2024;75(1):10–8. 10.5603/imh.98244. 38647055

[24] Szafran-Dobrowolska J, Grubman-Nowak M, Renke M, Jeżewska M. The psychosocial burden and stress coping strategies among seafarers. Int Marit Health. 2023;74(2):122–8. 10.5603/IMH.2023.0018. 37417846

[25] World Health Organization (WHO). Constitution of the World Health Organization. Geneva: World Health Organization, 1946.

[26] Pleban D, Kowalski P, Młynarczyk M, Orysiak J, Pawlak A, Warszewska-Makuch M, et al. Questionnaire for subjective assessment of working conditions on vessels. Bezpieczeństwo Pr Nauk Prakt. 2024;13–7. Polish. 10.54215/BP.2024.12.25.Pleban.

[27] Burr H, Berthelsen H, Moncada S, Nübling M, Dupret E, Demiral Y, et al. The third version of the *Copenhagen Psychosocial Questionnaire*. Saf Health Work. 2019;10(4):482–503. 10.1016/j.shaw.2019.10.002. 31890332 PMC6933167

[28] Goldberg DP, Hillier VF. A scaled version of the General Health Questionnaire. Psychol Med. 1979;9(1):139–45. 10.1017/s0033291700021644. 424481

[29] Polskie standardy jakości realizacji badań rynku i opinii społecznej [Internet]. Warsaw: Organizacja Firm Badania Opinii i Rynku [cited 2025 May 20]. Available from: https://www.pkjpa.pl/wp-content/uploads/2023/06/PKJPA_Standardy_12062023.pdf.

[30] Cohen J. Statistical power analysis for the behavioral sciences. 2nd ed. Hillsdale (NJ): Lawrence Erlbaum Associates; 1988.

[31] Fritz CO, Morris PE, Richler JJ. Effect size estimates: Current use, calculations, and interpretation. J Exp Psychol Gen. 2012;141(1):2–18. 10.1037/a0024338. 21823805

[32] Oldenburg M, Felten C, Hedtmann J, Jensen HJ. Physical influences on seafarers are different during their voyage episodes of port stay, river passage and sea passage: A maritime field study. PLoS One. 2020;15(4):e0231309. 10.1371/journal.pone.0231309. 32267889 PMC7141673

[33] Forsell K, Eriksson H, Järvholm B, Lundh M, Andersson E, Nilsson R. Work environment and safety climate in the Swedish merchant fleet. Int Arch Occup Environ Health. 2017;90(2):161–8. 10.1007/s00420-016-1180-0. 27815725 PMC5263194

[34] Jo D, Koh CK. Perceived hazardous physical work environments and job-related affective well-being of navy officers aboard the Republic of Korea Navy ships and submarines in South Korea. BMJ Mil Health. 2023;169(1):e29–33. 10.1136/bmjmilitary-2020-001702. PMC10176382 33593751

[35] Akamangwa N. Working for the environment and against safety: How compliance affects health and safety on board ships. Saf Sci [Internet]. 2016;87:131–43. 10.1016/j.ssci.2016.03.027.

